# The Story of the Insane from Year to Year

**Published:** 1889-08-31

**Authors:** 


### The Story of the Insane from Year to Year.

Asylum Reports.

### CITY OF LONDON ASYLUM.

This is the thirty-third annual report of the asylum for the City. On the last day of the year it contained 434 patients, being a decrease of 10 on the numbers for 1887. The recoveries reached the very creditable percentage of 51·25, and Dr. White attributes this high percentage to the employment and amusement of the patients. Doubtless, these are extremely valuable agents in the treatment of insanity, and we hope that there will be no falling off in the recovery rate for 1889, but a high percentage one year is very apt to mean a low one in the year following. The death-rate is low, being only 5·3, and post-mortem examination was made in every instance. The weekly cost of maintenance is 11s. 8d.

### MIDDLESEX COUNTY ASYLUM AT HANWELL.

It is just forty years since at this asylum Conolly joined in the crusade against the mechanical restraint of the insane. He here followed the example set in a much smaller institution by Gardiner Hill, and placed the non-restraint system on a basis so firm that nothing can ever seriously shake it. At that time the asylum contained about 800 patients, and it steadily increased its numbers until on the last day of December, 1888, there were 1,890 under treatment. During the year, 312 patients were admitted; 204 were discharged, and 109 died. The recoveries stand at 26·28 per cent., and the deaths at 5·76. The visitors' report is an unusually interesting one, and we think the following paragraph on pensions well worth reprinting: "The inducement of becoming in due course eligible for superannuation allowance has operated most strongly in retaining the services of experienced and consequently valuable servants. Fully aware that a feeling inimical to pensions is nowadays becoming more and more prevalent, the committee believe that in a lunatic asylum there exists no occupation where these rewards for good and faithful services are more needed and deserved." These are not mere words, as we notice in the report that two of the officers and several of the attendants received pensions. The weekly cost of maintenance is 9s. 4d. per head.

### BELFAST DISTRICT ASYLUM.

The town, or should we say city, of Belfast has a population of 200,000; and the district asylum takes in the insane patients from the county of Antrim and the town of Carrickfergus, as well as those of the city. At the close of 1888 there were 652 patients in residence, and the year's statistics show that 183 cases were admitted; 132 were discharged and 26 died. We find that 50 per cent. were discharged and that 4 per cent. died, the former being decidedly high and the latter low. Of the deaths four were owing to thoracic affections and 14 to brain diseases. Post-mortem examinations were made in only three instances. Of the admissions, hereditary influences account for 18 and intemperance for 29. Dr. Merrick points out that in 50 per cent. of the admissions the disease had lasted more than three months, and three were suffering from general paralysis, which Dr. Merrick remarks is unusual, "as for years there were no cases admitted labouring under this disease."

### BANSTEAD ASYLUM.

This is one of the Middlesex asylums, but it is situated in the county of Surrey. On the last day of the year it contained 2,003 patients. The admissions reached 559, the discharges 233, and the deaths 321. The recoveries were 33·63 per cent., and the deaths were 16·2, being one of the highest, if not the highest, we have yet met with, and is accounted for by the fact that general paralysis caused death in 71 instances and phthisis in 104. Esquirol said he could have written the history of the French Revolution from the delusions of his patients, and Dr. Shaw says that "the recent Whitechapel tragedies have had a very determining character upon the nature of the delusions both in men and women admitted from the East End of London." It is praiseworthy that Dr. Shaw has omitted beer from the diet-table of the non-workers among the men, and has substituted tea for afternoon beer on the women's side of the house; but we question whether he was right in reducing the animal food without putting in some other nitrogenous element. The weekly rate is 9s.

